# Left atrial volume as risk marker: is minimum volume superior to maximum volume?

**DOI:** 10.1093/ehjci/jeae136

**Published:** 2024-05-23

**Authors:** Otto A Smiseth, Camilla K Larsen, Einar Hopp

**Affiliations:** Institute for Surgical Research, Division of Cardiovascular and Pulmonary Diseases, Oslo University Hospital Rikshospitalet and Faculty of Medicine, University of Oslo, N-0027 Oslo, Norway; Institute for Surgical Research, Division of Cardiovascular and Pulmonary Diseases, Oslo University Hospital Rikshospitalet and Faculty of Medicine, University of Oslo, N-0027 Oslo, Norway; Institute for Surgical Research, Division of Cardiovascular and Pulmonary Diseases, Oslo University Hospital Rikshospitalet and Faculty of Medicine, University of Oslo, N-0027 Oslo, Norway; Division of Radiology and Nuclear Medicine, Oslo University Hospital, Oslo, Norway

**This editorial refers to ‘Comparative prognostic importance of measures of left atrial structure and function in non-ischaemic dilated cardiomyopathy’, by D.J. Hammersley *et al.*, https://doi.org/10.1093/ehjci/jeae080.**

Parameters of left atrial (LA) structure and function are used as biomarkers in a variety of cardiovascular conditions, including heart failure. The most widely used parameter is LA maximum volume, normalized for body surface area (LAVImax), and more recently LA reservoir strain was introduced as a measure of LA function. Due to the availability and relatively low cost, echocardiography is the standard modality for measuring LA volume and strain. In this issue of the journal, D.J. Hammersley *et al*.^1^ who evaluated cardiac structure and function by magnetic resonance imaging (CMR), challenge this approach by showing that LA minimum volume (LAVImin) was superior to LAVImax as a risk predictor. Furthermore, they concluded that LA strain had no additional value as a predictor when LA volume was included in the risk assessment.

In a prospective study of 580 patients with non-ischaemic dilated cardiomyopathy and sinus rhythm, Hammersley *et al*.^1^ used CMR to compare different measures of LA structure and function as risk predictors. The authors are to be commended for applying CMR, which is the most accurate modality to image cardiac structure, in such a large population to address an issue that is relevant to clinical practice. Their primary endpoint was a composite of cardiovascular death or nonfatal major heart failure events (cardiac transplantation, left ventricular assist device implantation, or heart failure hospitalization). The study also included several secondary endpoints. Over a median follow-up of 7.4 years, 103 patients (18%) met the primary endpoint. Parameters of LA structure and function included LAVImax, LAVImin, LA emptying fraction, LA reservoir strain, and LA pump strain (‘booster strain’). Each of these measures of LA structure and function was associated with the primary endpoint. The addition of each LA metric to a baseline model containing the same prognostic covariates improved model discrimination, with LAVImin providing the greatest improvement. Measures of LA strain did not enhance model discrimination above LA volumetric measures. The authors conclude that LAVImin was the best prognostic indicator in multivariable risk models and that LA strain did not incrementally improve risk prediction beyond LA volumes.

The observation by Hammersley *et al*.^1^ that LAVImin was superior to LAVImax as a risk predictor is consistent with previous studies which have used 2D or 3D echocardiography to measure LA volumes.^2–5^ Taken together, these studies showed that LAVImin was slightly better than LAVImax as a marker of elevated LV filling pressures and as a predictor of cardiovascular outcome. In these echocardiographic studies, however, interobserver variability was significantly larger for LAVImin than for LAVImax.^2–4,6^ This may reflect suboptimal signal-to-noise ratio when using echocardiography to image a small chamber in the far field of the image.

Not surprisingly, inter- and intra-observer variability for LAVImin by CMR in the study by Hammersley *et al*.^1^ was at the level of variability for measurement of LAVImax and less than with previous studies using echocardiography.^2–4,6^ Still, the incremental value of LAVImin in the study by Hammersley *et al*.^1^ was relatively small.

When Hammersley *et al*.^1^ conclude that LA strain was not incremental to volume parameters for risk prediction, one should be aware that one of their volume parameters, LA emptying fraction is an equivalent of LA reservoir strain. This was reflected in a very strong correlation between LA emptying fraction and LA reservoir strain (*r* = 0.92). Therefore, their conclusion that LA strain did not incrementally improve risk prediction beyond LA volumes is not transferable to echocardiographic imaging which usually includes just one volume parameter, (LAVImax) which is often reported together with LA reservoir strain.

When it comes to the assessment of LV filling pressure, LA reservoir and pump strains are superior to LAVImax as markers of LV filling pressure.^7–9^ In a recent study that compared a combination of LAVImax and LV mass by CMR to an echocardiographic multiparameter approach^10^ for assessment of LV filling pressure, echocardiography was superior.^11^ Potentially, more comprehensive CMR imaging with the use of LA strain or LA emptying fraction combined with LAVImin can improve CMR-based assessment of LV filling pressure.^12,13^

Currently, clinicians are somewhat overwhelmed by an increasing number of available echocardiographic parameters, and some may not be supported by strong data on clinical utility. When considering introducing novel parameters in cardiac diagnostics, one should search for a mechanistic rationale for why it should work, and therefore, in this case, try to understand why LAVImin should be superior to LAVImax. A potential mechanism can be that the two LA volume parameters respond differently to LV dysfunction. Since LA minimum volume is closely coupled to LV diastolic pressure and stiffness, which represent LA afterload, LA minimum volume tends to increase when heart failure progresses. This will be accompanied by an increase in LA maximum volume, but to a smaller degree since LV systolic dysfunction leads to a reduction in LA reservoir volume.^9,14^ Because LA maximum volume equals the sum of LA minimum volume and reservoir volume, there will be a tendency to less increase in LAVImax than in LAVImin in failing hearts. This proposed mechanism should be explored in future studies.

In conclusion, as shown by Hammersley *et al*.^1^ in patients with dilated cardiomyopathy using CMR to measure LA volumes, LAVImin was slightly better than LAVImax as a predictor of adverse cardiovascular outcome.^1^ This is consistent with several echocardiographic studies which have observed a minor improvement in risk prediction with LAVImin but with significantly weaker reproducibility than for LAVImax. More extensive validation of clinical utility and better reproducibility favours using LAVImax instead of LAVImin as a parameter of choice for echocardiography. With regards to CMR, studies in different heart failure phenotypes are required to determine whether a small improvement in risk prediction with LAVImin is clinically meaningful.

## Data Availability

No new data were generated or analysed in support of this research.
